# Inherited cardiomyopathies and sports participation

**DOI:** 10.1007/s12471-018-1079-3

**Published:** 2018-02-08

**Authors:** A. Zorzi, A. Pelliccia, D. Corrado

**Affiliations:** 1 Department of Cardiac, Thoracic and Vascular Sciences, University of Padova, Padova, Italy; 2 Institute of Sports Medicine and Science, Rome, Italy

**Keywords:** Athletes, Cardiomyopathy, Exercise prescription, Sports cardiology, Sudden cardiac death

## Abstract

Competitive sports activity is associated with an increased risk of sudden cardiovascular death in adolescents and young adults with inherited cardiomyopathies. Many young subjects aspire to continue competitive sport after a diagnosis of cardiomyopathy and the clinician is frequently confronted with the problem of eligibility and the request of designing specific exercise programs. Since inherited cardiomyopathies are the leading cause of sudden cardiovascular death during sports performance, a conservative approach implying disqualification of affected athletes from most competitive athletic disciplines is recommended by all the available international guidelines. On the other hand, we know that the health benefits of practicing recreational sports activity can overcome the potential arrhythmic risk in these patients, provided that the type and level of exercise are tailored on the basis of the specific risk profile of the underlying cardiomyopathy. This article will review the available evidence on the sports-related risk of sudden cardiac death and the recommendations regarding eligibility of individuals affected by inherited cardiomyopathies for sports activities.

**Fig. a:**
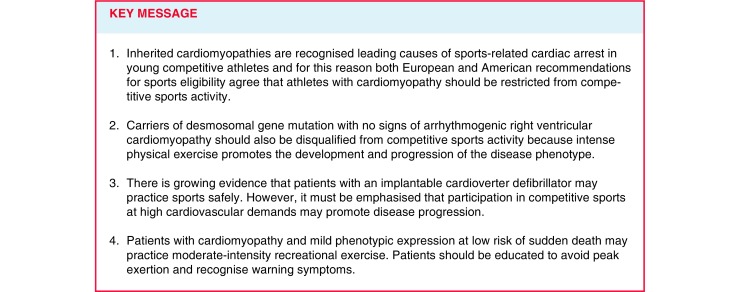
Trends of sudden death for cardiomyopathies among athletes in the Veneto region of Italy after implementation of a national preparticipation screening programme. Adapted with permission from Corrado et al. [1]

## Introduction

**Fig. 1:**
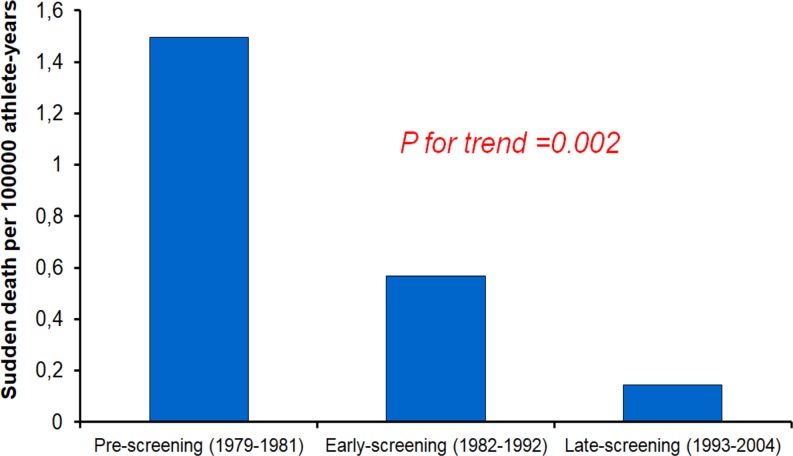
Trends of sudden death for cardiomyopathies among athletes in the Veneto region of Italy after implementation of a national preparticipation screening programme. Adapted with permission from Corrado et al. [1]

Competitive sports activity is associated with an increased risk of sudden cardiovascular death (SCD) in adolescents and young adults with clinically silent cardiovascular disorders [2]. Inherited cardiomyopathies are recognised leading causes of sports-related cardiac arrest in young competitive athletes, with hypertrophic cardiomyopathy (HCM) accounting for one-third of fatal cases in the USA and arrhythmogenic right ventricular cardiomyopathy for approximately one-fourth in Italy [3–5]. Preparticipation evaluation may allow the identification of asymptomatic athletes who have potentially lethal cardiomyopathies and protect them from the risk of SCD through restriction from competitive sports (Fig. 1; [6, 7]). However, many affected athletes aspire to continue practicing leisure-time sports activities or at least maintain a physically active lifestyle despite their non-eligibility to engage in competitive sports.

This article will review the available data on the sports-related risk of SCD and the recommendations regarding the eligibility to engage in sports activity, either competitive or recreational, of individuals affected by inherited cardiomyopathies. We will focus on major inherited cardiomyopathies which have been implicated as leading causes of SCD during sport activity and/or require a specific clinical work-up for differential diagnosis with physiologic remodelling of the athlete’s heart. We refer for sports participation with inherited channelopathies to Panhuyzen et al. in this same issue [8].

## Hypertrophic cardiomyopathy

Hypertrophic cardiomyopathy, the most common inherited heart muscle disease, is caused by mutations of genes encoding for sarcomeric proteins. The phenotype of HCM is characterised by left ventricular (LV) hypertrophy, either symmetric or asymmetric, which occurs in the absence of abnormal loading conditions. The diagnosis traditionally relies on the demonstration by echocardiography of otherwise unexplained diffuse or segmental LV wall and/or septal thickening ≥15 mm; a lower cut-off value (≥13 mm) is used in the context of family members of affected individuals, especially if they are mutation carriers [9, 10]. Cardiac magnetic resonance imaging may provide additional diagnostic and prognostic information given its ability to better detect segmental LV hypertrophy in some regions, such as anterior free wall, posterior septum or apex, which are not visualised adequately by echocardiography, to provide more accurate wall thickness measurements and to identify areas of intramyocardial late gadolinium enhancement/fibrosis. Affected patients are at an increased risk of ventricular arrhythmias/SCD, heart failure, and thromboembolism [11].

**Fig. 2:**
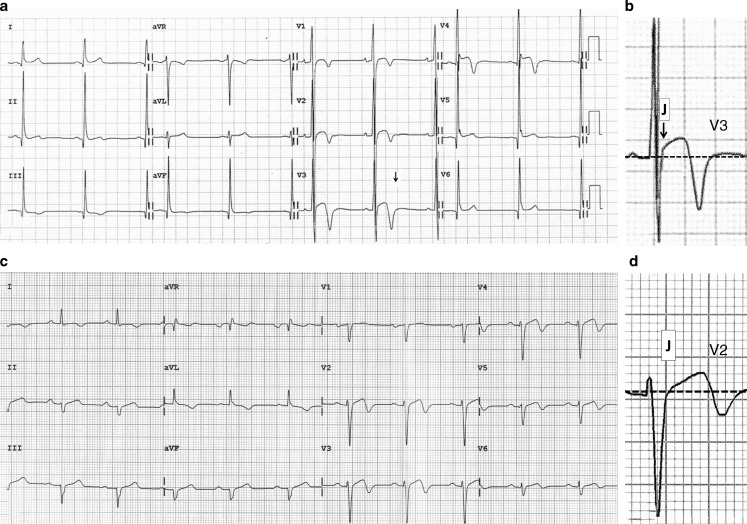
Anterior T‑wave inversion in healthy athletes and patients with hypertrophic cardiomyopathy. Electrocardiographic (ECG) tracing (**a**) and close-up of lead V3 (**b**) of a 21-year-old healthy athlete with anterior T‑wave inversion confined to V1–V4. Note that negative T waves in V3–V4 are preceded by J‑point elevation and ST-segment elevation (suggesting early repolarisation variant) and there are no associated ECG abnormalities. ECG tracing (**c**) and close-up of lead V2 (**d**) of a 32-year-old patient with hypertrophic cardiomyopathy showing anterior T‑wave inversion. Negative T waves in V2–V4 are not preceded by J‑point elevation. Moreover, T‑wave inversions extend also to lateral leads and left axis deviation is also present. Modified with permission from Calore et al. [16]

**Fig. 3:**
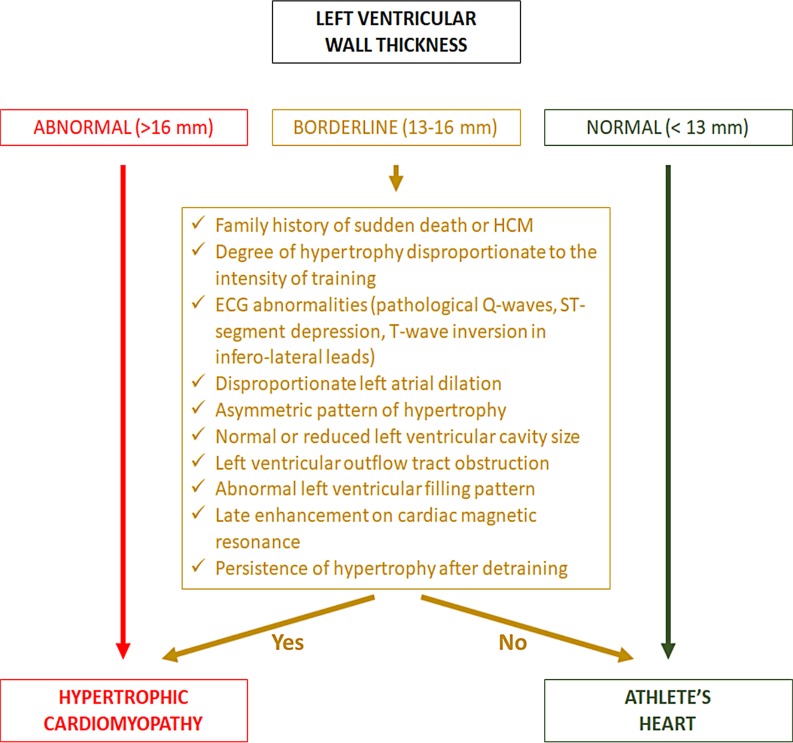
Differential diagnosis between athlete’s heart and hypertrophic cardiomyopathy. A subset of athletes exhibits an increase in left ventricular wall thickness that falls in the ‘grey zone’ of overlap between physiologic adaptation to exercise and pathologic hypertrophy. In these cases, the presence of positive family history of sudden cardiac death, cardiac arrest or inherited cardiac disease, electrocardiographic changes, echocardiographic abnormalities or late enhancement at cardiac magnetic resonance imaging suggests an underlying hypertrophic cardiomyopathy. On the other hand, symmetrical left ventricular hypertrophy with concomitant left ventricular dilatation compatible with gender (male > female), ethnicity (African/Afro-Caribbean > Caucasian) and intensity of training with no other abnormal features is suggestive of athlete’s heart

Left ventricular hypertrophy may develop as a consequence of heart adaption to physical exercise (athlete’s heart) and is particularly prominent in athletes involved in endurance sports activities, such as rowing and cycling [12], but maximal wall thickness rarely exceeds 13 mm [13–15]. A minority of highly trained athletes, particularly males of African/Afro-Caribbean descent, may exhibit more pronounced LV hypertrophy (13–16 mm), which requires an accurate clinical work-up for differential diagnosis with HCM [13–15]. In addition, in African/Afro-Caribbean athletes the electrocardiogram (ECG) often demonstrates a variant of the anterior early repolarisation pattern characterised by J‑point/ST-segment elevation and T‑wave inversion in the anterior precordial leads (V1 to V4) which may raise the suspicion of an underlying cardiomyopathy (Fig. 2; [16, 17]). A LV wall thickness >16 mm should be considered diagnostic of HCM even in highly trained athletes, irrespective of ethnicity [18, 19]. In athletes with LV hypertrophy falling into the ‘grey zone’ (13–16 mm), differential diagnosis between HCM and athlete’s heart is based on several parameters including imaging features (cavity size, distribution of hypertrophy, outflow tract obstruction, mitral valve abnormalities, diastolic function, and late enhancement at cardiac magnetic resonance imaging with a characteristic pattern of patchy involvement [20]) and ECG findings (ST-segment depression, T‑wave inversion in infero-lateral leads, pathological Q waves and conduction disturbances) (Fig. 3; [18, 19]).

**Fig. 4:**
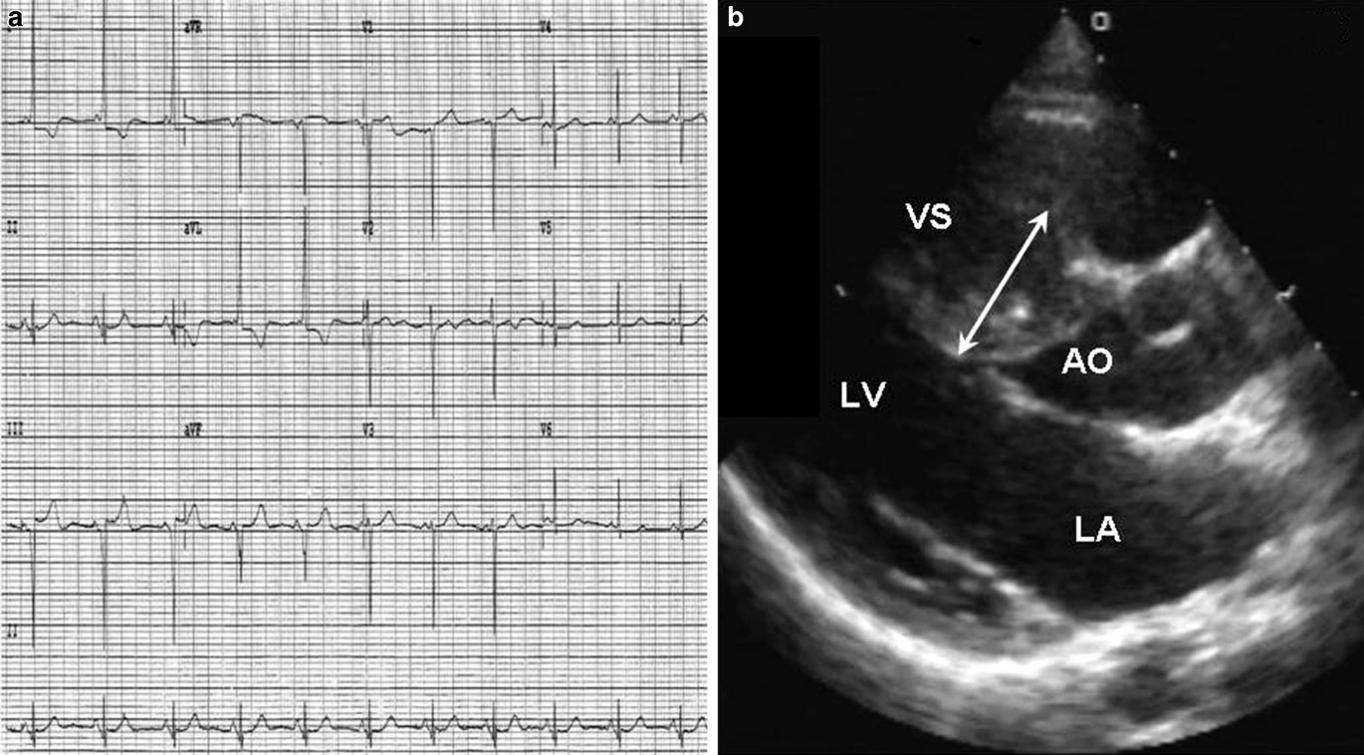
Electrocardiographic and echocardiographic findings in a 15-year-old male soccer player with hypertrophic cardiomyopathy. The electrocardiogram shows T‑wave inversion in lateral leads (I and aVL) and pathological Q‑wave (duration >25% of the height of the ensuing R‑wave) in inferior leads (III and aVF) (**a**). The echocardiogram shows an asymmetric left ventricular hypertrophy with maximal septal thickness of 31 mm (**b**). (*VS* ventricular septal, *LV* left ventricle, *LA* left atrium, *AO* aorta). Reproduced with permission from Migliore et al. [28]

Hypertrophic cardiomyopathy is one of the leading causes of SCD among athletes in the USA where it has been reported to account for more than one third of fatal cases [3, 4]. By contrast, in European studies HCM is reported to cause less than 10% of SCDs ([5, 21]; Table 1). Besides ethnic and genetic differences between the populations, this discrepancy may be explained by the preventive identification of HCM through systematic preparticipation screening including ECG, which is abnormal in up to 90% of athletes with HCM (Fig. 4; [1, 22–24]). ECG screening is common practice in most European countries and is compulsory in Italy, but it is not routinely performed in the USA.

**Table 1:** Main studies reporting the causes of cardiac arrest/sudden death in the athletes

Ref	Study period	Region	Population	Results (in %)
Corrado, JACC 2003 [5]	1979–1999	Veneto, Italy	SD among athletes 12–35 years old	*Cardiovascular SD*: 86
				CAD: 22
				ARVC: 14
				Myocarditis: 12
				Mitral valve prolapse: 10
				Conduction system disease: 10
				HCM: 9
				Aortic rupture: 5
				*Non cardiovascular SD*: 14
				*Unexplained SD*: 8
Corrado, JAMA 2006 [1]	1979–2004	Veneto, Italia	SD among 2,938,730 athletes 12–35 years old	*SD for structural heart disease among athletes*:
				Cardiomyopathy: 25
				CAD: 20
				Coronary anomalies: 13
				Myocarditis: 13
				Mitral valve prolapse: 11
				Conduction system disease: 7
			SD among 33,205,370 non-athletes 12–35 years old	*SD for structural heart disease among non-athletes*:
				Cardiomyopathy: 31
				CAD: 20
				Myocarditis: 15
				Conduction system disease: 9
				Mitral valve prolapse: 7
				Coronary anomalies: 5
Maron, Circulation, 2009 [3]	2001–2006	USA	SD among 10,700,000 athletes 13–25 years old	Unexplained: 34 HCM: 24 Coronary anomalies: 11 Possible HCM: 5
De Noronha, Heart, 2009 [25]	1996–2008	United Kingdom	Athletes with SD referred to the National Heart and Lung Institute and Royal Brompton Hospital pathology dpt.	Structurally normal heart: 23
				Idiopathic LV hypertrophy: 31
				ARVC: 14
				HCM: 11
				Idiopathic LV fibrosis: 6
				Coronary anomalies: 5
Harmon, Circulation Arrhythm Electrophysiol, 2014 [26]	2004–2008	USA	SD among National Collegiate Athletic Association athletes 17–24 years old	Unexplained 31
				Coronary anomalies 14
				Idiopathic LV hypertrophy: 8
				Aortic dissection: 8
				Myocarditis: 8
				Dilated cardiomyopathy: 8
				CAD: 5
Finocchiaro, J Am Coll Cardiol, 2016 [21]	1994–2014	United Kingdom	Athletes with SD referred to St. George’s University of London Cardiac Pathology dpt	Unexplained: 42
				Idiopathic LV hypertrophy/fibrosis: 16
				ARVC: 13
				HCM: 6
				Myocarditis: 5
Grani, Eur J Prev Cardiol, 2016 [27]	1999–2010	Switzerland	Exercise-related SD 10–39 years old	CAD: 28 HCM: 14 Unexplained: 13 Valvular: 8 Coronary anomalies: 7 ARVC: 7 Idiopathic myocardial fibrosis: 7 Aortic dissection: 7 Dilated cardiomyopathy: 6

*ARVC* arrhythmogenic right ventricular cardiomyopathy, *CAD* coronary artery disease, *HCM* hypertrophic cardiomyopathy, *LV* left ventricular, *SD* sudden death, *WPW* Wolff-Parkinson-White syndrome

High intensity competitive sports may promote life-threatening ventricular arrhythmias even in the absence of traditional risk factors and there is no evidence that algorithms developed to predict the risk of SCD in the general population of patients with HCM can be extrapolated to athletes. Accordingly, both European and American recommendations for sports eligibility agree that athletes with HCM should be restricted from competitive sports activity. Instead, participation in disciplines at low cardiovascular demand (such as bowling, golf and brisk walking) may be allowed to asymptomatic patients with mild LV hypertrophy (wall thickness <20 mm), no ventricular arrhythmias at exercise testing or ambulatory ECG monitoring and negative family history for SCD ([29, 30]; Table 2).

**Table 2:** Eligibility to competitive sports participation in athletes with cardiomyopathies based on the 2005 European Society of Cardiology and the 2017 American Heart Association/American College of Cardiology recommendations

Cardiomyopathy	Phenotype	Characteristics	Recommendations
HCM	High risk	Definitive diagnosis of HCM with symptoms, moderate to severe hypertrophy, ventricular arrhythmias or family history of sudden death	No competitive sports
	Low risk	Asymptomatic athletes with mild left ventricular hypertrophy, no ventricular arrhythmias and no sudden death in 1^st^ degree relatives	Only sports at low cardiovascular demands
	Healthy gene carrier	Carrier of pathogenetic mutation with no signs of disease	All competitive sports
ARVC	All phenotypes	Definitive diagnosis of ARVC	No competitive sports
	Healthy gene carrier	Carrier of pathogenetic mutation with no signs of disease	Only sports at low cardiovascular demand
DCM	High risk	Definitive diagnosis of DCM with symptoms, moderate to severe left ventricular dysfunction, ventricular arrhythmias or family history of sudden death	No competitive sports
	Low risk	Asymptomatic athletes with mild left ventricular dysfunction, no ventricular arrhythmias and no sudden death in 1^st^ degree relatives	Only sports at low cardiovascular demand
	Healthy gene carrier	Carrier of pathogenetic mutation with no signs of disease	All competitive sports
LVNC	High risk	Athletes with LVNC and symptoms, systolic dysfunction or ventricular arrhythmias	No competitive sports
	Low risk	Asymptomatic athletes with LVNC, normal systolic function and no ventricular arrhythmias	Competitive sport may be considered^a^
	Healthy gene carrier	Carrier of pathogenetic mutation with no signs of disease	All competitive sports

*ARVC* arrhythmogenic cardiomyopathy, dilated cardiomyopathy, *HCM* hypertrophic cardiomyopathy, *LVNC* left ventricular noncompaction

^a^According to the 2017 American Heart Association/American College of Cardiology guidelines

**Table 3:** Advised leisure-time exercise programmes in cardiomyopathy patients

Preliminary evaluation	High-risk features	Examples of disciplines	General advice
1) History 2) Physical examination 3) Electrocardiogram (ECG) 4) Maximal exercise testing 5) 24-hour ambulatory ECG monitoring 6) Echocardiography This evaluation should be repeated at regular time-intervals (e. g. annually)	1) Symptoms 2) Family history for sudden cardiac death 3) Moderate to severe echocardiographic abnormalities 4) Abnormal blood pressure response or ST modifications during exercise testing 5) Clinically relevant arrhythmias	*All patients:* Brisk walking, jogging Cycling (flat course) Golf Swimming^a^ Stationary bicycle Treadmill Low-intensity weights *Patients with NO high-risk features:* Team sport Running Tennis Cross country skiing	1) Start with a warm-up period 2) Increase gradually the length and intensity of exercise session 3) If possible, monitor heart rate and maintain 60–80% of the maximal heart rate obtained at exercise testing (or estimated as 220-age) 4) Avoid high-intensity interval exercise 5) Finish with a cool-down period 6) Reintegrate hydrosaline leaks

In general, activities to be prudentially avoided in patients with cardiomyopathies are those performed in dangerous environments or associated with increased risk for life if syncope occurs

^a^The possibility of impaired consciousness occurring during water-related activities should be taken into account with respect to the clinical profile of the individual patient

## Arrhythmogenic cardiomyopathy

Arrhythmogenic right ventricular cardiomyopathy (ARVC) is an inherited cardiomyopathy characterised by progressive loss of myocytes and replacement by fibro-fatty tissue, which creates the substrate for life-threatening ventricular arrhythmias. In its classical form the phenotypic manifestations predominantly involve the right ventricle, but biventricular and left-dominant variants exist. Molecular genetic studies showed that ARVC is a genetic disorder resulting from defective desmosomal proteins, most often with an autosomal dominant pattern of inheritance [31, 32].

The clinical manifestations of the disease usually occur between the second and forth decade of life and consist of ECG depolarisation and repolarisation changes, typically localised in the right precordial leads (V1–V3/V4), structural abnormalities, such as global or regional dilatation/dysfunction of the right ventricle and, most importantly, ventricular arrhythmias with a left bundle branch block morphology, ranging from isolated premature ventricular beats to sustained ventricular tachycardia, which can degenerate into ventricular fibrillation [33, 34].

The diagnosis of ARVC requires a combination of different criteria from various categories such as 1) histopathological abnormalities at endomyocardial biopsy; 2) morpho-functional abnormalities consisting of regional right ventricular wall motion abnormalities (regional akinesia, dyskinesia or bulging) *plus *right ventricular dilatation or global right ventricular dysfunction; 3) depolarisation abnormalities such as epsilon waves, delayed S‑wave upstroke in V1–V3 and late potentials on signal-averaged ECG; 4) T-wave inversion; 5) ventricular arrhythmias and 6) positive family history of ARVC or SCD and/or identification of a pathogenetic mutation by molecular genetic testing [35].

Arrhythmogenic cardiomyopathy has been demonstrated to be the leading cause of SCD in athletes of the Veneto region of Italy (14% of cases), whereas in the USA it accounts for only 6% of cases ([3–5]; Table 1). This finding may depend on the experience of the pathologist or coroner who performs post-mortem investigation as ARVC is rarely associated with cardiomegaly and may spare the left ventricle, so that the affected heart may be erroneously diagnosed as normal. Of note, in a British study on SCD in athletes, whose hearts were referred for post-mortem to a tertiary centre, the prevalence of ARVC was similar (13%) to that found in the Italian series [21].

**Fig. 5:**
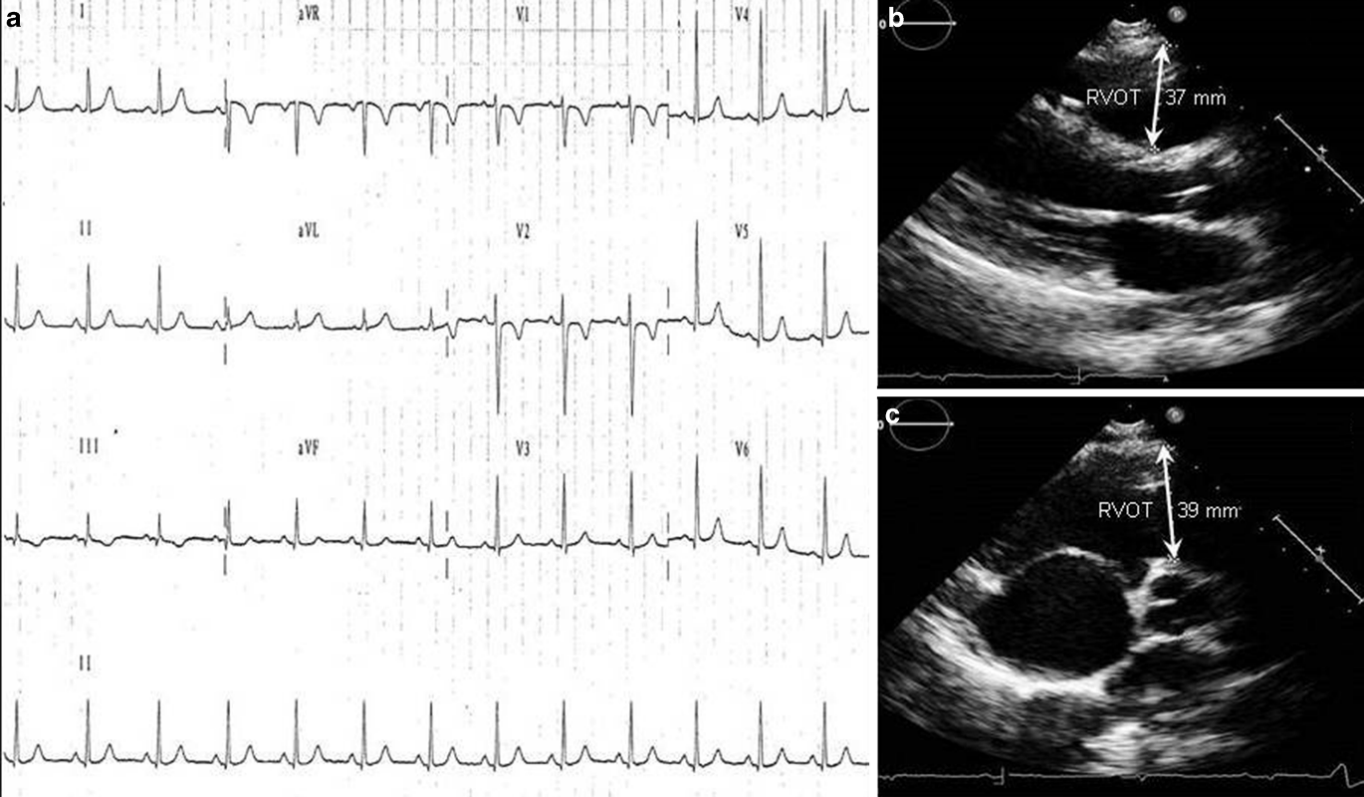
Electrocardiographic and echocardiographic findings in a 14-year-old male soccer player with right ventricular dominant (classic phenotype) arrhythmogenic cardiomyopathy. The electrocardiogram shows T‑wave inversion in right precordial leads (V1–V2) (**a**). The echocardiogram reveals right ventricular dilatation (right ventricular outflow tract diameter of 39 mm on end-diastolic parasternal short-axis view) (**b**) and right ventricular dysfunction (akinesia of right ventricular outflow tract and posterobasal, subtricuspidal regions) (not shown). Reproduced with permission from Migliore et al. [28]

**Fig. 6:**
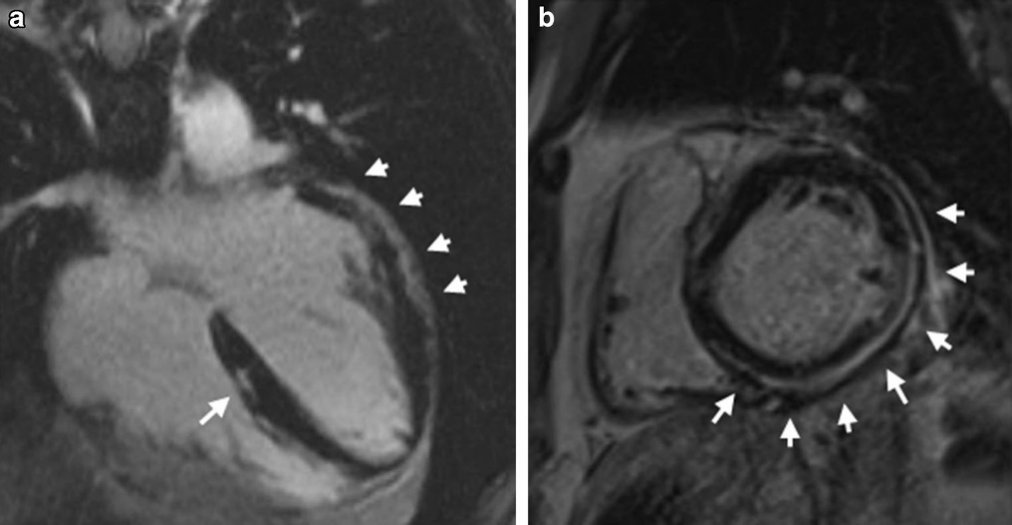
Representative example of a left dominant arrhythmogenic cardiomyopathy. Post-contrast cardiac magnetic resonance findings of an asymptomatic 23-year-old female carrying a desmoplakin gene mutation. Four-chamber (**a**) and short-axis (**b**) views showing late gadolinium enhancement mostly involving the subepicardial layer of the posterolateral left ventricular wall at mid-basal level (*white arrows*), in the absence of other morpho-functional ventricular abnormalities. The electrocardiogram and echocardiogram of this patient were normal. Reproduced with permission from Zorzi et al. [40]

The ‘left dominant’ variant of ARVC is characterised by an early and predominant LV involvement, as a result of a specific genetic background [36]. At variance with the classic ‘right-dominant’ variant (Fig. 5), the power of traditional investigations including routine ECG and standard echocardiography for the diagnosis of ‘left-dominant’ ARVC is limited because repolarisation abnormalities and left ventricular systolic dysfunction, either regional or global, are observed in a minority of affected patients. The reason is that the fibro-fatty scarring process predominantly involves the sub-epicardial and mid-mural myocardial layers, which contribute less to ventricular contraction, and the disease lesion can be only identified in the form of late gadolinium enhancement using contrast-enhanced cardiac magnetic resonance imaging (Fig. 6; [37]). Not surprisingly, the incidence of SCD in patients with the classic ARVC variant has markedly decreased since the introduction of ECG preparticipation screening, while the difficult-to-diagnose ‘left dominant’ variant is now an increasingly emergent substrate at post-mortem [38, 39].

**Fig. 7:**
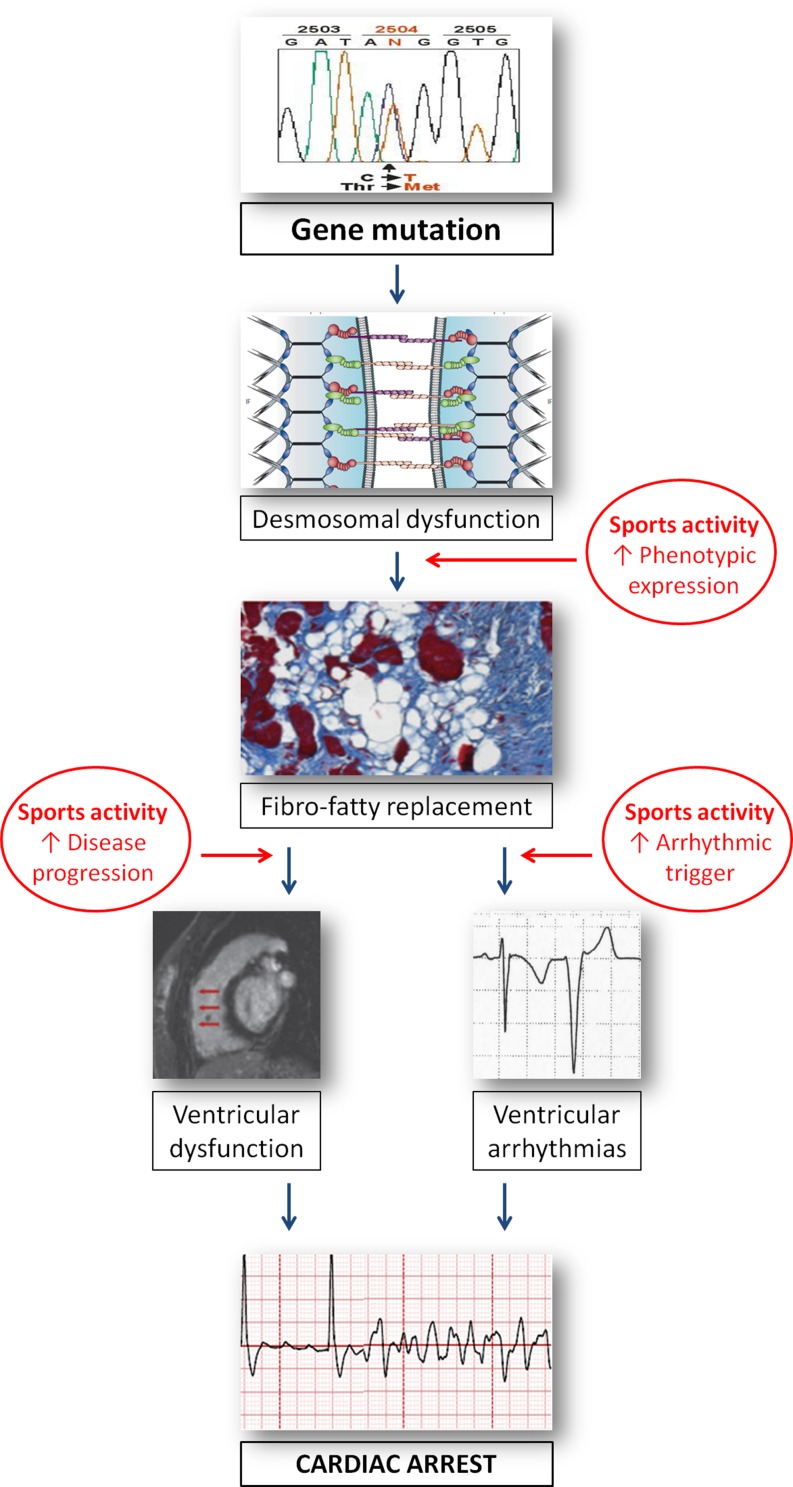
Schematic representation of ARVC course from desmosomal gene mutation to phenotypic expression and cardiac arrest due to ventricular fibrillation. Sports activity may promote development of phenotypic expression, accelerate disease progression and trigger life-threatening ventricular arrhythmias. Reproduced with permission from Corrado et al. [47]. (*ARVC* arrhythmogenic right ventricular cardiomyopathy)

Competitive sports activity increases the risk of SCD by 5‑fold in adolescent and young adults with ARVC [5]. Sports has also been implicated as the most important environmental factor promoting ARVC progression and worsening of the disease arrhythmic substrate (Fig. 7). Actually, genetically determined impairment of cell-to-cell adhesion may predispose to myocyte detachment and death, mostly under condition of increased mechanical wall stress such as that occurring during competitive sports activity [41]. Kirchhof et al. [42] demonstrated that in heterozygous plakoglobin-deficient mice, endurance training accelerated the development of right ventricular abnormalities and ventricular arrhythmias. James et al. [43] and Saberniak et al. [44] confirmed in clinical studies that endurance sports and intense physical exercise increase age-related penetrance, risk of ventricular tachyarrhythmias and occurrence of heart failure in ARVC desmosomal gene carriers. Ruwald et al. [45] reported that the absolute risk of malignant arrhythmic events in ARVC patients who participated in competitive sports reached 61% at 40 years of age. On the other hand, early identification of affected patients by preparticipation screening and disqualification from competitive sports activity offers the potential to prevent SCD [1]. Accordingly, both European and American recommendations for sports eligibility in patients with heart diseases and the International Task Force consensus document on ARVC treatment agree that restriction from competitive sports activity of patients with ARVC should be regarded as a therapeutic measure aimed to reduce the risk of SCD (Table 2; [29, 30, 46]).

## Dilated cardiomyopathy and left ventricular noncompaction

Dilated cardiomyopathy (DCM) is characterised by dilatation and systolic dysfunction of the left ventricle which is genetically determined in approximately one third of cases. Up to 40 genes have been identified, which affect proteins of a wide variety of cellular structures such as the sarcomere, the nuclear envelope, the cytoskeleton, the sarcolemma and the intercellular junction [48]. Differential diagnosis between athlete’s heart and DCM may be challenging as a sizeable proportion of athletes, mostly those engaged in endurance sports activities, exhibit an enlarged left ventricle and a subset of them show a mild reduction in ejection fraction, i. e. between 45 and 55% [49, 50]. The diagnosis of DCM is suggested by the presence of the following features: depressed systolic function with ejection fraction below 45%, associated regional wall motion abnormalities, concomitant right ventricular dysfunction, positive family history of SCD, cardiac arrest or inherited cardiac disease, and evidence of ECG abnormalities, such as T‑wave inversion or intraventricular conduction defects, complex ventricular arrhythmias at 24-hour Holter monitoring or stress testing and late gadolinium enhancement at contrast-enhanced cardiac magnetic resonance. In borderline cases, demonstration of a significant (>10%) increase of the ejection fraction during exercise echocardiography indicates a preserved contractile reserve and may support the diagnosis of athlete’s heart versus DCM [51].

The American Heart Association consensus statement on definition and classification of cardiomyopathies considers the left ventricular noncompaction (LVNC) as a genetic heart muscle disorder [52], while a European Society of Cardiology consensus document reports it as ‘unclassified cardiomyopathy’ [53]. This inconsistency in the classification of LVNC can be explained by the uncertain nature of the disease, either a separate condition or a phenotypic variant of other cardiomyopathies. Pathogenic mutations have been identified in up to 41% of the affected subjects, in a significant proportion of cases (29%) as sarcomere gene mutations [54, 55]. Although current imaging criteria for the diagnosis of LVNC are based on the ratio between the compact and non-compact LV layers, the thickness of the non-compacted myocardium or the number of trabeculations, these morphologic features are non-specific and can be observed in up to 8% of highly trained athletes (particularly of African/Afro-Caribbean ethnicity). These morphologic changes have been interpreted as a result of the chronic increase in the LV preload occurring during intense training and exercise. In this regard, hypertrabeculation has also been observed in healthy pregnant women (with complete resolution or marked reduction of trabeculation after delivery) or in patients with chronic anaemia [56–58]. Hypertrabeculation is rarely associated with LV systolic dysfunction, ECG repolarisation abnormalities, or late gadolinium enhancement, suggesting a ‘LVNC cardiomyopathy’ [58]. Hence, hypertrabeculation in isolation can be part of the adaptive heart changes to exercise and should not be considered a cardiac disease [59].

Dilated cardiomyopathy is an uncommon cause of SCD in young competitive athletes. In the Veneto region of Italy, it accounted for 4% of fatalities with cardiovascular origin [5], while in the USA, Maron et al. found it in 2.5% [3] and Harmon et al. in 8% [26] of SCD victims. Finocchiaro et al. reported a 1% prevalence of fatal DCM in British athletes [21]. It is noteworthy that none of the above-mentioned studies reported isolated LVNC as a cause of SCD in athletes (Table 1).

Athletes affected by either DCM or LVNC are considered at significant risk of SCD during exercise, and both European and American recommendations agree that affected athletes are not eligible to engage in competitive sports activity. Conversely, asymptomatic athletes with isolated LVNC, i. e. with a normal LV systolic function, no ECG abnormalities and/or ventricular arrhythmias, and no evidence of late enhancement at cardiac magnetic resonance imaging can participate in competitive sports ([29, 30]; Table 2).

## Healthy gene carriers

At present, there is no scientific evidence demonstrating that competitive sports activity increases the risk of disease development or sudden death in athletes who carry a pathogenetic mutation but who do not show any sign of phenotypic manifestation (i. e., genotype-positive/phenotype-negative individuals). However, ARVC associated with desmosome gene mutation constitutes an important exception: in this condition, physical exercise and sports activity have been implicated as a key environmental factor for promoting the development and progression of the disease phenotype, both in animal models [42] and in clinical studies on gene mutation carriers [43–45].

## Implantable cardioverter defibrillator (ICD) and sports activity

Patients with an ICD are traditionally considered not eligible to engage in competitive sports activity, except for disciplines characterised by a low cardiovascular demand which do not expose to the risk of traumatic damage of the device [60]. However, there is growing evidence that ICD patients may practice sports safely. Lampert et al. [61] reported on 372 US athletes with ICDs (age 10–60 years) participating in either competitive (*N* = 328) or dangerous (*N* = 44) sports. During a median follow-up of 31 months, 46 athletes had appropriate therapies (25 during physical exertion) and 29 inappropriate interventions (25 during physical exertion). There were no deaths, resuscitated cardiac arrests or arrhythmia related injuries during sports activity. Freedom from lead malfunction was 97% at 5 years and 90% at 10 years from ICD implant. The authors concluded that their results on efficacy and safety of ICD therapy in the athletic population did not differ significantly from available data in non-athletic ICD patients and, thus, do not support restriction of sports activity for ICD patients. In line with these new data, the most recent recommendations of the American Heart Association regarding the eligibility and disqualification of athletes with cardiovascular abnormalities to engage in competitive sports stated that competitive sports may be allowed in selected athletes with an ICD [62]. The new subcutaneous ICD system appears a valuable therapeutic option for young patients with cardiomyopathy to avoid the risk of transvenous lead damage. However, we should emphasise that the reasons for competitive sports restriction in young patients with cardiomyopathy who have received an ICD for the prevention of sudden cardiac death go beyond the increased risk of unsuccessful shock, inappropriate interventions, injury to the patient, or damage of the device. In fact, as discussed above for patients with ARVC, sports participation may play a key role in the heart muscle disease progression, worsening of the arrhythmic substrate and adverse outcome.

## Recreational sports activity

Recreational sports include a wide range of physical activities, from modest to vigorous in intensity, performed either in a regular or inconsistent basis not requiring systematic training or pursuit of excellence, nor involving the same psychological pressure to surpass other participants, which is characteristic of competitive sports.

Although there is a paucity of scientific data regarding the risk of SCD in patients with cardiomyopathies engaging in recreational sports activity, it is likely that a certain degree of risk will remain. However, it seems reasonable to state that this potential risk of exercise should not entirely deprive cardiomyopathy patients of the many benefits offered by regular exercise [63]. A recent study in a small cohort of HCM patients with mild phenotype suggested that moderate-intensity physical activity may provide benefits in terms of fitness with no significant arrhythmic risk [64]. Ruwald et al. [45] reported that the absolute risk of ventricular tachyarrhythmias/death at 40 years after birth was high (33%) in ARVC patients practicing recreational sports, but did not significantly differ from that of physically inactive ARVC patients (22%).

With regard to the prescription of recreational exercise programmes in patients with a diagnosis of cardiomyopathy, the following criteria which characterise a ‘low risk’ of SCD should be taken into account: 1) no symptoms; 2) negative family history for SCD; 3) ‘mild’ structural abnormalities; 4) normal or near-normal response to exercise (i. e. no ST-segment depression and normal increase of blood pressure during stress testing); 5) absence of clinically relevant arrhythmias at exercise testing or during 24-hour ambulatory ECG monitoring. When all these conditions are fulfilled, the physician can reassure the patient with respect to his ability and safety to sustain a regular exercise programme; if one or more risk criteria are present, only low intensity physical activities should be prescribed (Table 3).

Patients should be educated to start an exercise session with a warm-up period and specifically avoid peak exertion, characterised by high-intensity interval exercise. At the end of the session, an appropriate cool-down period is also recommended. The clinician should teach patients to take control over the level of exertion by assessing heart rate with commercially available devices. Exercise in extremely adverse environmental conditions, including very hot, humid, or extremely cold weather should be avoided. Patients should be carefully informed on the specific risk profile of their disease, the potentially dangerous sports activities, mostly in the presence of a history of syncope, and the warning symptoms that may occur in association with exercise. Finally, the availability of an automated external defibrillator in the athletic field should be considered as an additional ‘back-up’ measure to prevent SCD.

## References

[1] Corrado D, Basso C, Pavei A, Michieli P, Schiavon M, Thiene G (2006). Trends in sudden cardiovascular death in young competitive athletes after implementation of a preparticipation screening program. JAMA.

[2] Corrado D, Zorzi A (2017). Sudden death in athletes. Int J Cardiol.

[3] Maron BJ, Doerer JJ, Haas TS, Tierney DM, Mueller FO (2009). Sudden deaths in young competitive athletes: analysis of 1866 deaths in the United States, 1980–2006. Circulation.

[4] Van Camp SP, Bloor CM, Mueller FO, Cantu RC, Olson HG (1995). Nontraumatic sports death in high school and college athletes. Med Sci Sports Exerc.

[5] Corrado D, Basso C, Rizzoli G, Schiavon M, Thiene G (2003). Does sports activity enhance the risk of sudden death in adolescents and young adults?. J Am Coll Cardiol.

[6] Corrado D, McKenna WJ (2007). Appropriate interpretation of the athlete’s electrocardiogram saves lives as well as money. Eur Heart J.

[7] Corrado D, Biffi A, Basso C, Pelliccia A, Thiene G. 12-lead ECG in the athlete: physiological versus pathological abnormalities. Br J of Sports Med 2009;43:669-76. 10.1136/bjsm.2008.054759 19734501

[8] Panhuyzen-Goedkoop NM, Wilde AAM (2018). Heart.

[9] Elliott PM, Anastasakis A, Borger MA (2014). 2014 ESC Guidelines on diagnosis and management of hypertrophic cardiomyopathy: the Task Force for the Diagnosis and Management of Hypertrophic Cardiomyopathy of the European Society of Cardiology (ESC). Eur Heart J.

[10] Gersh BJ, Maron BJ, Bonow RO (2011). 2011 ACCF/AHA guideline for the diagnosis and treatment of hypertrophic cardiomyopathy: a report of the American College of Cardiology Foundation/American Heart Association Task Force on Practice Guidelines. Circulation.

[11] Marian AJ, Braunwald E (2017). Hypertrophic cardiomyopathy: genetics, pathogenesis, clinical manifestations, diagnosis, and therapy. Circ Res.

[12] Mihl C, Dassen WR, Kuipers H (2008). Cardiac remodelling: concentric versus eccentric hypertrophy in strength and endurance athletes. Neth Heart J.

[13] Pelliccia A, Maron BJ, Spataro A, Proschan MA, Spirito P (1991). The upper limit of physiologic cardiac hypertrophy in highly trained elite athletes. N Engl J Med.

[14] Basavarajaiah S, Boraita A, Whyte G, Wilson M, Carby L, Shah A, Sharma S (2008). Ethnic differences in left ventricular remodeling in highly–trained athletes relevance to differentiating physiologic left ventricular hypertrophy from hypertrophic cardiomyopathy. J Am Coll Cardiol.

[15] Basavarajaiah S, Wilson M, Whyte G, Shah A, McKenna W, Sharma S (2008). Prevalence of hypertrophic cardiomyopathy in highly trained athletes: relevance to pre–participation screening. J Am Coll Cardiol.

[16] Calore C, Zorzi A, Sheikh N, Nese A, Facci M, Malhotra A, Zaidi A, Schiavon M, Pelliccia A, Sharma S, Corrado D (2016). Electrocardiographic anterior T‑wave inversion in athletes of different ethnicities: differential diagnosis between athlete’s heart and cardiomyopathy. Eur Heart J.

[17] Papadakis M, Carre F, Kervio G, Rawlins J, Panoulas VF, Chandra N, Basavarajaiah S, Carby L, Fonseca T, Sharma S (2011). The prevalence, distribution, and clinical outcomes of electrocardiographic repolarization patterns in male athletes of African/Afro-Caribbean origin. Eur Heart J.

[18] Caselli S, Maron MS, Urbano-Moral JA, Pandian NG, Maron BJ, Pelliccia A (2014). Differentiating left ventricular hypertrophy in athletes from that in patients with hypertrophic cardiomyopathy. Am J Cardiol.

[19] Pelliccia A, Caselli S, Sharma S (2017). European Association of Preventive Cardiology (EAPC) and European Association of Cardiovascular Imaging (EACVI) joint position statement: recommendations for the indication and interpretation of cardiovascular imaging in the evaluation of the athlete’s heart. Eur Heart J.

[20] Patel AR, Kramer CM (2017). Role of cardiac magnetic resonance in the diagnosis and prognosis of nonischemic cardiomyopathy. JACC Cardiovasc. Imaging.

[21] Finocchiaro G, Papadakis M, Robertus JL (2016). Etiology of sudden death in sports: insights from a united kingdom regional registry. J Am Coll Cardiol.

[22] Sheikh N, Papadakis M, Ghani S (2014). Comparison of electrocardiographic criteria for the detection of cardiac abnormalities in elite black and white athletes. Circulation.

[23] Corrado D, Basso C, Schiavon M, Thiene G (1998). Screening for hypertrophic cardiomyopathy in young athletes. N Engl J Med.

[24] Harmon KG, Zigman M, Drezner JA (2015). The effectiveness of screening history, physical exam, and ECG to detect potentially lethal cardiac disorders in athletes: a systematic review/meta-analysis. J Electrocardiol.

[25] de Noronha SV, Sharma S, Papadakis M, Desai S, Whyte G, Sheppard MN (2009). Aetiology of sudden cardiac death in athletes in the United Kingdom: a pathological study. Heart.

[26] Harmon KG, Drezner JA, Maleszewski JJ, Lopez-Anderson M, Owens D, Prutkin JM, Asif IM, Klossner D, Ackerman MJ (2014). Pathogeneses of sudden cardiac death in national collegiate athletic association athletes. Circ Arrhythm Electrophysiol.

[27] Grani C, Chappex N, Fracasso T, Vital C, Kellerhals C, Schmied C, Saguner AM, Trachsel LD, Eser P, Michaud K, Wilhelm M (2016). Sports-related sudden cardiac death in Switzerland classified by static and dynamic components of exercise. Eur J Prev Cardiol.

[28] Migliore F, Zorzi A, Michieli P (2012). Prevalence of cardiomyopathy in Italian asymptomatic children with electrocardiographic T‑wave inversion at preparticipation screening. Circulation.

[29] Pelliccia A, Fagard R, Bjornstad HH (2005). Recommendations for competitive sports participation in athletes with cardiovascular disease: a consensus document from the Study Group of Sports Cardiology of the Working Group of Cardiac Rehabilitation and Exercise Physiology and the Working Group of Myocardial and Pericardial Diseases of the European Society of Cardiology. Eur Heart J.

[30] Maron BJ, Udelson JE, Bonow RO, Nishimura RA, Ackerman MJ, Estes NA, Cooper LT, Link MS, Maron MS (2015). Eligibility and disqualification recommendations for competitive athletes with cardiovascular abnormalities: task force 3: hypertrophic cardiomyopathy, arrhythmogenic right ventricular cardiomyopathy and other cardiomyopathies, and myocarditis: a scientific statement from the American Heart Association and American College of Cardiology. J Am Coll Cardiol.

[31] Corrado D, Link MS, Calkins H (2017). Arrhythmogenic right ventricular cardiomyopathy. N Engl J Med.

[32] Zorzi A, Rigato I, Bauce B, Pilichou K, Basso C, Thiene G, Iliceto S, Corrado D (2016). Arrhythmogenic right ventricular cardiomyopathy: risk stratification and indications for defibrillator therapy. Curr Cardiol Rep.

[33] Corrado D, Basso C, Thiene G (1997). Spectrum of clinicopathologic manifestations of arrhythmogenic right ventricular cardiomyopathy/dysplasia: a multicenter study. J Am Coll Cardiol.

[34] Basso C, Corrado D, Marcus F, Nava A, Thiene G (2009). Arrhythmogenic right ventricular cardiomyopathy. Lancet.

[35] Marcus FI, McKenna WJ, Sherrill D (2010). Diagnosis of arrhythmogenic right ventricular cardiomyopathy/dysplasia: proposed modification of the task force criteria. Circulation.

[36] Sen-Chowdhry S, Syrris P, Prasad SK, Hughes SE, Merrifield R, Ward D, Pennell DJ, McKenna WJ (2008). Left-dominant arrhythmogenic cardiomyopathy: an under-recognized clinical entity. J Am Coll Cardiol.

[37] Zorzi A, Perazzolo Marra M, Rigato I (2016). Nonischemic left ventricular scar as a substrate of life-threatening ventricular arrhythmias and sudden cardiac death in competitive athletes. Circ Arrhythm Electrophysiol.

[38] d’Amati G, De Caterina R, Basso C (2016). Sudden cardiac death in an Italian competitive athlete: pre-participation screening and cardiovascular emergency care are both essential. Int J Cardiol.

[39] Basso C, Rizzo S, Pilichou K, Corrado D, Thiene G (2014). Why arrhythmogenic cardiomyopathy is still a major cause of sudden death in competitive athletes despite preparticipation screening?. Circulation.

[40] Zorzi A, Rigato I, Pilichou K (2016). Phenotypic expression is a prerequisite for malignant arrhythmic events and sudden cardiac death in arrhythmogenic right ventricular cardiomyopathy. Europace.

[41] Thiene G, Nava A, Corrado D, Rossi L, Pennelli N (1988). Right ventricular cardiomyopathy and sudden death in young people. N Engl J Med.

[42] Kirchhof P, Fabritz L, Zwiener M (2006). Age- and training-dependent development of arrhythmogenic right ventricular cardiomyopathy in heterozygous plakoglobin-deficient mice. Circulation.

[43] James CA, Bhonsale A, Tichnell C, Murray B, Russell SD, Tandri H, Tedford RJ, Judge DP, Calkins H (2013). Exercise increases age-related penetrance and arrhythmic risk in arrhythmogenic right ventricular dysplasia/cardiomyopathy-associated desmosomal mutation carriers. J Am Coll Cardiol.

[44] Saberniak J, Hasselberg NE, Borgquist R, Platonov PG, Sarvari SI, Smith HJ, Ribe M, Holst AG, Edvardsen T, Haugaa KH (2014). Vigorous physical activity impairs myocardial function in patients with arrhythmogenic right ventricular cardiomyopathy and in mutation positive family members. Eur J Heart Fail.

[45] Ruwald AC, Marcus F, Estes NA, Link M, McNitt S, Polonsky B, Calkins H, Towbin JA, Moss AJ, Zareba W (2015). Association of competitive and recreational sport participation with cardiac events in patients with arrhythmogenic right ventricular cardiomyopathy: results from the North American multidisciplinary study of arrhythmogenic right ventricular cardiomyopathy. Eur Heart J.

[46] Corrado D, Wichter T, Link MS (2015). Treatment of arrhythmogenic right ventricular cardiomyopathy/dysplasia: an international task force consensus statement. Circulation.

[47] Corrado D, Zorzi A (2015). Arrhythmogenic right ventricular cardiomyopathy and sports activity. Eur Heart J.

[48] Favalli V, Serio A, Grasso M, Arbustini E (2016). Genetic causes of dilated cardiomyopathy. Heart.

[49] Pelliccia A, Culasso F, Di Paolo FM, Maron BJ (1999). Physiologic left ventricular cavity dilatation in elite athletes. Ann Intern Med.

[50] Abergel E, Chatellier G, Hagege AA, Oblak A, Linhart A, Ducardonnet A, Menard J (2004). Serial left ventricular adaptations in world-class professional cyclists: implications for disease screening and follow-up. J Am Coll Cardiol.

[51] Galderisi M, Cardim N, D’Andrea A (2015). The multi-modality cardiac imaging approach to the Athlete’s heart: an expert consensus of the European Association of Cardiovascular Imaging. Eur Heart J Cardiovasc Imaging.

[52] Elliott P, Andersson B, Arbustini E (2008). Classification of the cardiomyopathies: a position statement from the European Society Of Cardiology Working Group on Myocardial and Pericardial Diseases. Eur Heart J.

[53] Maron BJ, Towbin JA, Thiene G, Antzelevitch C, Corrado D, Arnett D, Moss AJ, Seidman CE, Young JB (2006). Contemporary definitions and classification of the cardiomyopathies: an American Heart Association Scientific Statement from the Council on Clinical Cardiology, Heart Failure and Transplantation Committee; Quality of Care and Outcomes Research and Functional Genomics and Translational Biology Interdisciplinary Working Groups; and Council on Epidemiology and Prevention. Circulation.

[54] Hoedemaekers YM, Caliskan K, Michels M, Frohn-Mulder I, van der Smagt JJ, Phefferkorn JE, Wessels MW, ten Cate FJ, Sijbrands EJ, Dooijes D, Majoor-Krakauer DF (2010). The importance of genetic counseling, DNA diagnostics, and cardiologic family screening in left ventricular noncompaction cardiomyopathy. Circ Cardiovasc Genet.

[55] Probst S, Oechslin E, Schuler P, Greutmann M, Boye P, Knirsch W, Berger F, Thierfelder L, Jenni R, Klaassen S (2011). Sarcomere gene mutations in isolated left ventricular noncompaction cardiomyopathy do not predict clinical phenotype. Circ Cardiovasc Genet.

[56] Gati S, Papadakis M, Papamichael ND, Zaidi A, Sheikh N, Reed M, Sharma R, Thilaganathan B, Sharma S (2014). Reversible de novo left ventricular trabeculations in pregnant women: implications for the diagnosis of left ventricular noncompaction in low-risk populations. Circulation.

[57] Gati S, Papadakis M, Van Niekerk N, Reed M, Yeghen T, Sharma S (2013). Increased left ventricular trabeculation in individuals with sickle cell anaemia: physiology or pathology?. Int J Cardiol.

[58] Gati S, Chandra N, Bennett RL (2013). Increased left ventricular trabeculation in highly trained athletes: do we need more stringent criteria for the diagnosis of left ventricular non-compaction in athletes?. Heart.

[59] Caselli S, Attenhofer Jost CH, Jenni R, Pelliccia A (2015). Left ventricular noncompaction diagnosis and management relevant to pre-participation screening of athletes. Am J Cardiol.

[60] ElMaghawry M, Migliore F, Zorzi A, Bauce B, Leoni L, Bertaglia E, Iliceto S, Corrado D (2013). Implantable cardioverter-defibrillator therapy in athletes. Card Electrophysiol Clin.

[61] Lampert R, Olshansky B, Heidbuchel H (2013). Safety of sports for athletes with implantable cardioverter-defibrillators: results of a prospective, multinational registry. Circulation.

[62] Zipes DP, Link MS, Ackerman MJ, Kovacs RJ, Myerburg RJ, Estes NA (2015). Eligibility and disqualification recommendations for competitive athletes with cardiovascular abnormalities: task force 9: arrhythmias and conduction defects: a scientific statement from the American Heart Association and American College of Cardiology. Circulation.

[63] Maron BJ, Chaitman BR, Ackerman MJ (2004). Recommendations for physical activity and recreational sports participation for young patients with genetic cardiovascular diseases. Circulation.

[64] Saberi S, Wheeler M, Bragg-Gresham J (2017). Effect of moderate-intensity exercise training on peak oxygen consumption in patients with hypertrophic cardiomyopathy: a randomized clinical trial. JAMA.

